# Cancer patients’ insights and experience of palliative and supportive care in the UAE: a qualitative study

**DOI:** 10.3389/fonc.2026.1829844

**Published:** 2026-05-14

**Authors:** Khalifa Mohammad AlOlama, Salem Omar Alameri, Yasin Wissi, Waleed Mezher Alabdli, Hamad Hisham AlHammadi, Ahmed Gabr AlDhaheri, Mariam Ali AlNaqbi, Mahmoud Ahmed Elshafey, Nour Abushousha, Abdullah Humaid O. Al-Shamsi, Sarah Humaid O. Al-Shamsi, Mohammed Humaid O. Al-Shamsi, Ahmad Tariq Kalantar, Albesher Y.Alfoteih, Abdulrahman Mahmoud Basioni, Wessam Alkrad, Siddig Ibrahim Abdelwahab, Amal Al Balushi, Faryal Iqbal, Neil Arun Nijhawan, Humaid O. Al-Shamsi

**Affiliations:** 1 College of Medicine, Mohammed Bin Rashid University of Medicine and Health Sciences, Dubai, United Arab Emirates; 2 Barts and The London School of Medicine and Dentistry, Queen Mary University of London, London, United Kingdom; 3 RAK College of Medical Sciences, RAK Medical & Health Sciences University, Ras Al Khaimah, United Arab Emirates; 4 Emirates American School, Sharjah, United Arab Emirates; 5 School of Medicine, University College Dublin, Dublin, Ireland; 6 Burjeel Cancer Institute, Burjeel Medical City, Abu Dhabi, United Arab Emirates; 7 Health Research Centre, Jazan University, Jazan, Saudi Arabia; 8 Nursing Program, Oman College of Health Science, Muscat, Oman; 9 Emirates Oncology Society, Emirates Medical Association, United Dubai, United Arab Emirates; 10 Gulf Medical University, Ajman, United Arab Emirates; 11 Department of Medical Oncology, Dana-Farber Cancer Institute, Harvard Medical School, Boston, MA, United States; 12 College of Medicine, Ras Al Khaimah Medical and Health Sciences University, Ras Al Khaimah, United Arab Emirates; 13 College of Medicine, Gulf Medical University, Ajman, United Arab Emirates

**Keywords:** caregivers, communication, health services accessibility, neoplasms, pain management, palliative care, United Arab Emirates

## Abstract

**Introduction:**

The increasing prevalence of advanced cancer cases requiring palliative care highlights the growing need for palliative care as an essential component of comprehensive cancer management. However, despite rising global demand, awareness, access, and integration remain inadequately understood, particularly in rapidly developing and culturally diverse contexts such as the United Arab Emirates (UAE). Insights from such settings can enhance understanding of how palliative care is delivered, perceived, and adapted across diverse populations.

**Methods:**

This qualitative descriptive study explored the experiences and perceptions of cancer patients and their caregivers regarding palliative and supportive care services in a major oncology healthcare setting in the UAE. Semi-structured interviews were conducted with 10 participants at the Burjeel Cancer Institute in Abu Dhabi. Data were analyzed using thematic analysis.

**Results:**

Five interrelated themes were identified: “Awareness and Understanding of Palliative Care,” “Balancing Pain Relief and Medication Concerns,” “Cultural Expectations and Financial Considerations,” “Navigating the Palliative Care Experience,” and “Voices for Change: Improving Palliative Care.” Findings revealed a general lack of initial awareness about palliative care, with understanding often developing only after engagement with services. Challenges included communication difficulties, language barriers, and limited access to care outside the hospital setting. Cultural beliefs and family dynamics influenced decision-making, while insurance coverage played a key role in facilitating access. Despite these challenges, participants reported generally positive experiences with multidisciplinary care and symptom management.

**Discussion:**

This study contributes to the international literature by providing novel insights into palliative care delivery within a multicultural, high-resource yet culturally complex healthcare system. The findings highlight the need for enhanced public awareness, improved provider–patient communication, and stronger integration of palliative care within cancer services. Although limited by a small sample size and single-institution setting, the study offers transferable lessons for similar contexts. Further multicenter research is recommended to inform national-level strategies and improve equitable access to palliative care in the UAE.

## Introduction

Palliative care is an approach that improves the quality of life of patients and their families facing life-threatening illness by relieving physical, psychosocial, and spiritual suffering. It is delivered by a multidisciplinary team, and it can be integrated early alongside disease-directed therapies to optimize patient-centered outcomes. Early integration of palliative care enhances quality of life, reduces symptom burden, improves coping and prognostic awareness, and, in some cases, extends survival while decreasing aggressive end-of-life interventions (1–3).

Given these benefits, the global need for palliative care is rising rapidly, driven by population aging and the increasing burden of cancer and other non-communicable diseases. Projections estimate that the number of people experiencing serious health-related suffering will nearly double by 2060, with cancer accounting for a substantial share. Despite this growing demand, access to palliative care remains uneven and limited across regions, highlighting the importance of understanding how such services are experienced and implemented within healthcare contexts (4, 5).

In the United Arab Emirates (UAE), healthcare infrastructure has advanced significantly over the past two decades, with the establishment of modern cancer centers through international partnerships. Palliative and supportive care services have expanded from a limited base (primarily in Abu Dhabi and Dubai since 2007) to a modest but growing network across approximately four centers nationwide (6–9). Despite this progress, palliative care remains an evolving component of the healthcare system, and there is limited research capturing the lived experiences of patients and caregivers, particularly in relation to awareness, access, and cultural influences on care.

From an international perspective, examining these experiences is important, as the UAE represents a transitional, high resource, yet culturally complex healthcare setting. Insights from such contexts can contribute to a broader understanding of how palliative care is delivered, perceived, and adapted across diverse populations.

Therefore, this qualitative study aims to explore the perceptions and lived experiences of cancer patients and their caregivers regarding palliative and supportive care services in an oncology setting in the UAE, to address the following research questions:

1. How do cultural, financial, and linguistic factors influence experiences of palliative care?
2. What challenges do patients and caregivers face in accessing and utilizing palliative care services?
3. What recommendations do patients and caregivers suggest to improve palliative care services?

## Background

Palliative care has evolved from primarily end-of-life support to an essential, early integrated component of comprehensive cancer management. International evidence, including multiple randomized trials, demonstrates that timely specialist palliative care improves quality of life, alleviates physical and psychological symptoms, supports caregivers, enhances prognostic understanding, and can reduce unnecessary aggressive interventions (10, 11). Systematic reviews and guidelines, such as those from American Society of Clinical Oncology (ASCO), strongly recommend early integration for patients with advanced cancer to achieve better patient-centered outcomes (10).

The global burden of serious health-related suffering requiring palliative care is escalating. Recent projections indicate that by 2060, approximately 48 million people annually will die with serious health-related suffering, an 87% increase from 2016, with cancer as the leading driver (16.3 million cancer decedents experiencing such suffering). This rise is most pronounced in low- and middle-income countries and among older adults, underscoring the urgent need for scalable, context-appropriate palliative oncology models (4, 12).

Implementation varies widely across regions. In high-income settings, multidisciplinary palliative oncology teams are more established, yet disparities in timely referral and equitable access persist (13, 14).

In addition, common barriers affecting the implementation of the palliative care, including insufficient specialist training, regulatory restrictions on opioids and essential medications, limited-service integration into oncology care, cultural taboos around discussing death, and prognosis. Moreover, in Arab and Muslim-majority contexts, additional complexities arise from family-centered decision-making, religious perspectives on suffering, and preferences regarding information disclosure (15, 16). These factors highlight the need for context-specific research to better understand how palliative care is perceived and utilized across different cultural settings.

In the UAE, cancer care has progressed rapidly, with over 30 cancer centers and clinics now operating, and a national focus on reducing cancer mortality. Palliative care services, however, remain limited and concentrated in major cities, with services expanding slowly from the first public program in 2007 to a few additional private-sector providers. Existing UAE research has primarily examined healthcare providers’ or residents’ perspectives, revealing gaps in formal palliative care education, communication challenges, and emotional burdens on clinicians. In contrast, direct insights from cancer patients and caregivers, particularly regarding awareness, pain management, cultural and financial influences, service experiences, and improvement recommendations, are scarce (7, 17).

However, there is limited in-depth qualitative research exploring the experiences of patients and caregivers in the UAE, particularly regarding awareness, pain management, cultural influences, and access to care. Addressing this gap is essential to inform patient-centered service development and policy.

This study addresses a critical gap by exploring the lived experiences of cancer patients and caregivers within a major oncology center in the UAE. It provides transferable insights for similar transitional and multicultural healthcare systems in the Gulf region and beyond, while informing strategies for public education, healthcare provider training, service integration, and policy development to enhance equitable palliative oncology care (7).

## Methodology

### Study design

This study adopted a qualitative research design guided by a descriptive phenomenological approach (18), to explore the lived experiences of cancer patients and their caregivers regarding palliative care services in the UAE. The epistemological foundation of this study is rooted in phenomenology, which focused on how knowledge is constructed through individuals’ conscious experiences of phenomena (18, 19). Grounded on Husserlian descriptive (transcendental) phenomenology, this study assumes that reality can be understood through a description of participants lived experiences as they are perceived. Therefore, bracketing (epoche) was used to minimize the researcher’s prior bias and assumptions and to enhance methodological rigor (18, 19). Data were collected through in-depth interviews, transcribed verbatim, and analyzed systematically using Barun and Clarke’s framework (20). This inductive approach enabled identification of patterns and themes that reflect the essence of participants’ experiences.

### Participant recruitment and sampling

Participants were recruited through purposive sampling based on their experience with palliative care services. Eligible participants included adults (≥18 years) with advanced cancer receiving palliative care at Burjeel Cancer Institute (BCI), Abu Dhabi, who had previously discussed palliative care with their treating physicians. Family caregivers (≥18 years) of eligible patients were also invited to participate in patient nomination. Patient participants were classified as receiving palliative care by their oncology care team and were registered under the care of the palliative care department. Individuals were excluded if they were under 18 years of age or unable to participate in a conversational interview due to their medical condition.

Eligible patients and their caregivers were identified by the clinical team. Three authors (HOA, NAN, and WAK) introduced the study to potential participants during routine consultations. The BCI was purposively selected as the study site because it is one of the largest comprehensive cancer centers in the UAE, managing a high volume of oncology cases and hosting the country’s first dedicated palliative care service. All eligible patients who were approached agreed to participate, and no refusals were recorded. A total of 10 participants were enrolled, including five patients receiving palliative care and five family caregivers. Recruitment continued until data saturation was reached, defined as the point at which no new information or themes emerged (21, 22).

Interviews were conducted by trained medical students with prior experience in clinical communication and data collection and research, and co-investigators. The interviewers had no prior personal or clinical relationship with the participants before the study, and no pre-existing relationship influenced the data collection. The interviewers maintained a neutral, non-hierarchical stance throughout the interviews to minimize bias and ensure open and honest responses.

This study was conducted and reported in accordance with the COREQ checklist (Consolidated Criteria for Reporting Qualitative Research) to ensure methodological rigor and transparency (23) and is provided Top of Form as supplementary material.

### Data collection

Data were collected through face-to-face semi-structured interviews between August 7 and 14, 2024, by the medical students trained in qualitative research methods, using a semi-structured interview guide. Interviews lasted between 10 and 20 minutes and were conducted in private rooms (patient rooms). In accordance with participant preference, data were documented using detailed field notes during conducting the interview, rather than audio recordings.

An interview topic guide (**Table 1**) was developed to facilitate discussions, encouraging participants to share their personal experiences in their own words. The interview guide was developed based on clinical expertise and relevant literature (24, 25), covering key domains of palliative care, and was refined through team discussions and pilot testing. The guide was pilot-tested and refined for clarity and relevance. The phrasing and coherence of the questions were tested during the first interview, where two authors were present, and adjustments were made based on this initial feedback. The interviews were conducted either with the patient alone or in the presence of their family caregivers, depending on participants’ preferences. During the sessions, detailed notes were taken to document observations and reflections. At the start of each interview, demographic information such as age, gender, and nationality was collected to provide context for the participants. Each participant was interviewed once; no repeat interviews were report.

**Table 1:** Topic guide for palliative and supportive care interview.

**I. Demographic information** How old are you? What is your gender? What is your nationality?
**II. Diagnosis and treatment** What is your current or past diagnosis? *(If asking on behalf of a family member, inquire about the patient’s diagnosis).* What is the current stage of the disease? At what stage of your diagnosis were you offered palliative or supportive care? Please clarify. What symptoms led to your referral to palliative or supportive care? How long had you been experiencing these symptoms before being referred to palliative or supportive care? *(Now, days, weeks, months)* Do you believe that the referral and evaluation by palliative or supportive care have alleviated your symptoms? Please explain your experience. If you experienced a late referral to palliative and supportive care services, do you wish you had been referred earlier? Why or why not?
**III. Awareness and knowledge** Before your referral, were you aware of palliative or supportive care services? If so, how did you learn about them? How would you describe your understanding of palliative or supportive care before receiving these services? Do you feel you received enough information about palliative or supportive care from your healthcare providers? If yes, please provide details.
**IV. Experience with palliative and supportive care** Can you describe your experience with palliative and supportive care services in the UAE? What types of palliative and supportive care services have you received (e.g., physical, emotional, psychological, spiritual support, etc.)? How satisfied are you with the palliative and supportive care services you received? Please explain. Did you face any difficulties in accessing palliative and supportive care services? If yes, please tell me more about them. How did your family and friends participate in your palliative and supportive care?
**V. Multidisciplinary team and support** Did you receive care from a multidisciplinary team (e.g., doctors, nurses, social workers)? If yes, how do you rate the coordination and communication among the team members? Do you think the healthcare providers you met were well-trained in palliative and supportive care in your experience? Have you ever received palliative or supportive care in another country or institution? If so, how was your experience there compared to here? Did you receive psychological or emotional support as part of your palliative and supportive care? If yes, how beneficial was it?
**VI. Pain management and medications** Did you face any difficulties in accessing painkillers, especially opioids? If yes, please describe. How effective have painkillers and management strategies been for you in the current and past times? Do you have any concerns about using opioids for pain management? If yes, please provide details.
**VII. Cultural and financial aspects** Did you face any cultural challenges or resistance in accepting palliative and supportive care? If yes, please provide details. How did the financial cost of palliative and supportive care affect you and your family? Do you have insurance or other forms of coverage for these services? For example, home care or home services related to palliative and supportive care?
**VIII. Additional comments** What do you think can be improved in palliative and supportive care services in the UAE? Do you have any other comments or suggestions regarding palliative and supportive care?

Bolded text indicates major thematic sections of the interview topic guide.

The interviewers adopted an empathetic approach, beginning with open-ended, non-threatening questions to build rapport. They allowed participants sufficient time to respond, used active listening and probing questions for clarification, and avoided medical jargon. Communication impasses were managed through rephrasing questions in simpler terms and ensuring a comfortable, private environment to encourage open dialogue.

### Ethical approval and data management

The study received ethical approval from the Burjeel Institutional Review Board (BH/REC/072/24). Participation in this study was voluntary, and informed consent was obtained from participants prior to data collection. Participants were assured of confidentiality and anonymity, as well as their right to withdraw from the study at any time without penalty. No incentive was given to the participants. All data were securely stored and used solely for the research purpose. The lead author transcribed the interview field notes, and all transcripts were anonymized to protect participants’ identities. To reduce the burden on participants, especially those with limited life expectancy or varying literacy levels, the transcripts were not returned to them for validation.

### Data analysis

An inductive thematic analysis was conducted following Braun and Clarke’s six-phase framework (20). Initial codes were generated through line-by-line coding of transcripts. Codes were then reviewed and grouped based on similarities into subthemes, which were further refined into overarching themes. Two authors independently coded a subset of transcripts, compared codes, and iteratively developed themes and subthemes through discussion until consensus. No qualitative software was used; analysis was manual.

Trustworthiness was ensured following the criteria of Lincoln and Guba (1985) (26). Credibility was enhanced through investigator triangulation and peer debriefing. Dependability was supported by an audit trail of decisions. Confirmability was maintained via reflexivity (researchers’ positionality being documented). Transferability is supported by thick descriptions of context. Participant validation (member checking) was not conducted due to time constraints and participants’ advanced illness. Nevertheless, trustworthiness was maintained through alternative strategies as outlined above, in accordance with the criteria proposed by Lincoln and Guba (1985) (26).

## Results

### Study population

A total of 10 participants were included in this study, the majority of whom had advanced-stage cancer. Participants represented diverse demographic backgrounds, including both males and females, with ages ranging from 39 to 68 years, and different nationalities. The major types of cancer represented included breast, lung, colorectal, and gynecological cancers. Participant demographic data characteristics are presented in **Table 2**.

**Table 2:** Patient demographics and interview details.

Interview number	Participants present at interview	Participant identifier	Patient Age at recruitment, years	Patient’s gender	Patient’s nationality	Patient’s primary medical condition(s)
1	Patient	P-01	50	M	Emirati	Rectal Cancer (Stage IV)
2	Patient	P-02	52	F	Gaza – Palestine	Breast cancer four years ago, and currently pancreatic cancer
3	Family caregiver	F-01	60	F	Emirati	Endometrial Cancer (Stage IV)
4	Family caregiver	F-02	39	M	Egyptian	Rectal cancer (Stage IV)
5	Family caregiver	F-03	65	F	Gaza – Palestine	Endometrial cancer (Stage IV)
6	Family caregiver	F-04	68	F	Emirati	Metastatic fallopian tube cancer (Stage IV)
7	Family caregiver	F-05	67	F	Gaza – Palestine	Colorectal cancer (Stage IV)
8	Patient	P-03	58	F	Philippines	Right breast cancer and brain metastasis (Stage IV)
	Family caregiver	F-06	N/A	F	Philippines	N/A
9	Patient	P-04	57	M	Emirati	lung cancer (Stage IV)
10	Patient	P-05	54	M	Gaza- Palestinian	Brain tumor (Glioblastoma Grade IV)
	Companion	C-01	N/A	M	Gaza-Palestinian	N/A

F, female; M, male; N/A, not applicable.

### Qualitative findings

Participants’ experiences with palliative and supportive care revealed significant variability, highlighting the uniqueness of each patient’s journey and their diverse views on their condition and the care provided. Five interconnected themes emerged from the cancer patients’ insights and experiences of palliative and supportive care in the UAE.

- Theme 1: Awareness and Understanding of Palliative Care
- Theme 2: Balancing pain relief and medication concerns
- Theme 3: Cultural Expectations and Financial Considerations
- Theme 4: Navigating the Palliative Care Experience
- Theme 5: Voices for Change: Improving Palliative Care

#### Theme 1: awareness and understanding of palliative care

The Majority of participants demonstrated limited awareness of palliative care prior to referral, with understanding developing only after engagement with services. This delayed awareness reflects a potential gap in public health-related communication awareness about palliative care services.

As illustrated by one participant, palliative was perceived as a*”different world”*, (P-01, patient participant) where severe pain is relieved, allowing him to regain a sense of normalcy.

*Similarly, family caregiver noted “We didn’t know anything about it and what palliative care meant, but praise be to God, things became clearer”* (F-01, family caregiver).

Whereas one of the participants was already familiar with palliative care services due to previous experiences with a family member. One caregiver explained, *“We knew about them long time ago, because we brought our father before that”, …”They provide painkillers, so the patient doesn’t feel pain”* (F-04, family caregiver). This prior exposure provided a foundational understanding of palliative care, specifically the administration of pain relief.

However, even among those who had heard of palliative care, comprehension was often limited. As one participant stated, *“We heard of it but I didn’t know what it was”* (P-04), underscoring the distinction between awareness and meaningful understanding.

#### Theme 2: balancing pain relief and medication concerns

Pain management was the central of the participants’ experiences with palliative care. Most participants reported no difficulties in accessing painkillers, particularly within the hospital setting, compared to outside the hospital. As one participant shared,

“In the hospital, there are no difficulties; they give them to you, but when you take them by yourself from the pharmacy, it takes time. (P-01, patient participant)”.

*“Yes, we had difficulties receiving certain medications (Tramadol), but they gave it to us in the hospital and not outside of the hospital because of the censorship and policies” (P-05, C01, patient participant and patient companion)*.

While participants expressed their satisfaction with pain relief, concerns about medication use were also evident. For example, a caregiver shared, *“Yes, there were fears, especially days you see symptoms such as hallucinations and dizziness, but they reassured us that this was really temporary, and their words were correct” (F-01, family caregiver*), indicating the need for reassurance and clear communication regarding side effects.

In contrast, some participants reported inadequate pain control alongside concerns about over medication. One caregiver stated that “*The pain has worsened” …”Yes, I’m afraid they are giving too much”* (F-03, family caregiver). These findings illustrate the complexity of pain management, where effective relief must be balanced with patient and family concerns Theme.

#### Theme 3: cultural expectations and financial considerations

Cultural beliefs and family dynamics significantly influenced decision-making related to palliative care. Some participants describe initial resistance from the family regarding the palliative care treatment plan. As one caregiver shared, *“Yes, the family wanted to refuse my brother’s treatment and wanted him to go back to Egypt in which he refused” (F-02, family caregiver)*, indicating the tension between cultural expectations and clinical recommendations.

Financial considerations were generally mitigated by insurance coverage, which reduced the burden on patients and families. Participants frequently emphasized this aspect, noting *“the insurance covered everything regarding the palliative care service” (F-02, family caregiver)*, and *“We have Daman insurance; they cover it.” (P-03; F-06).* These findings indicated that while culture shape decision-making and acceptance, insurance and financial supports can facilitate access to care.

#### Theme 4: navigating the palliative care experience

Participants generally reported positive experience with palliative care services at BMC, particularly in terms of multidisciplinary team involvement and symptoms management. Improvements in comfort and daily functioning were commonly described. For example, participants stated, *“It helped with her sleeping (F-06; family caregiver)”and “I can sleep now” (P-03- patient participant)*, reflecting the tangible benefit of care.

Nevertheless, some participants reporting complex journeys across multiple healthcare system elsewere before accessing palliative care in the UAE. For example, one caregiver shared,

*“Initially, we went to Thailand, which was good, but the medication damaged her kidneys. Then we went to Singapore, which was also good, but the treatment didn’t work. We returned to the American Hospital in the UAE, but she had a brain hemorrhage. My sister told me that Burjeel offers good care, so we brought her there” (F-04, family care giver)*, emphasizing the role of the local services in meeting patients need after unsuccessful experiences elsewhere.

#### Theme 5: voices for change: improving palliative care

Participants identified several areas for improvement, primarily related to communication, staffing, and service responsiveness. Language barriers were a key concern, with the participant’s emphasizing the need for improvement and timely explanation in Arabic. For example, one participant stated, “Only if someone can explain to the patient what the doctor or nurse is saying. No one speaks Arabic, but the doctor explained it to me on the second day”… “Yes, better to be immediately so I can know” (P02- patient participant).

Similarly, another participant highlighted, “The problem is that the medical team do not speak Arabic, so there needs to be a translator” (P-05 patient participant), underscoring the importance of culturally and linguistically appropriate care.

The need for staffing and responsiveness was also raised. A caregiver noted, “There aren’t enough specialists (lack of specialization variety), and nurses take about 15 minutes to respond at night shifts specifically” (F-05, family caregiver)

**Figure 1:**
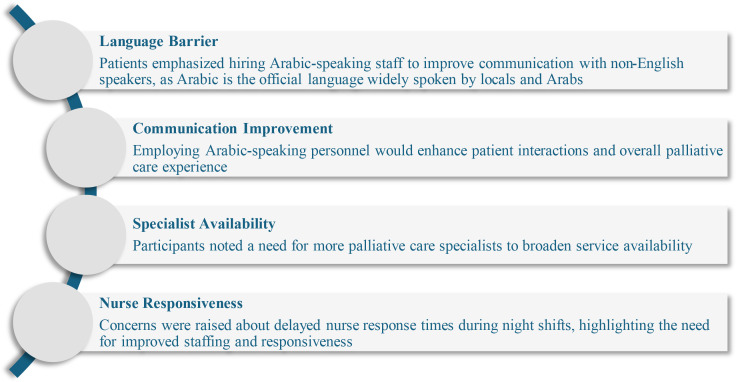
Key areas for improvement in palliative and supportive care services as suggested by participants.

Most patients reported overall satisfaction with palliative and supportive care services in the UAE. However, their feedback highlighted key areas for improvement, as shown in **Figure 1**.

## Discussion

Palliative care is an essential component of comprehensive cancer management, particularly with patients with advanced and complex illnesses (27). There is increasing interest in developing strategies to actively identify patients with palliative care needs within primary care settings (28–31). This study aimed to explore patients’ and caregivers’ lived experiences of palliative care in the UAE using an inductive qualitative approach. To the author’s knowledge, this is the first study using a semi-structured interview model to gather cancer patients’ insights and identify the challenges they currently face during their palliative care treatment in the UAE.

Similar to qualitative studies conducted elsewhere that used semi-structured interviews with advanced cancer patients, our participants reported significant challenges related to symptom burden, communication, and psychosocial support during palliative treatment. However, unlike many prior studies that primarily focused on primary care identification or Western healthcare contexts, our work highlights context-specific issues in the Middle East, including cultural factors, limited early integration of palliative services, and the hospital-centric nature of care delivery in the UAE (25, 32).

The findings demonstrate limited baseline awareness of palliative care, with understanding often developing only after direct engagement with services (Theme 1). This reflects a significant gap in public awareness and communication about palliative care services. Our findings showed that 30% of participants had no prior knowledge of palliative care, while most others developed an appreciation only after exposure during treatment.

These findings align with regional literature, which often reports limited awareness of palliative care in populations. In an assessment of the knowledge, beliefs, obstacles, and resources available for providing palliative care services in fifteen Middle Eastern countries, Silbermann et al. (33), identified several major barriers. These include a lack of palliative care beds and services, insufficient training for healthcare providers, limited community awareness, inadequate access to hospice services, and a shortage of time and personnel (34). Our observation that 30% of participants had no prior knowledge and that many only developed an appreciation after direct exposure mirrors these reported gaps in community awareness. However, unlike earlier regional studies that primarily highlighted systemic and provider-level barriers, our patient-centered data demonstrate that targeted education and improved provider communication can rapidly enhance understanding and perceived value. Furthermore, the role of family experiences in building prior knowledge underscores the cultural importance of familial involvement in healthcare decisions within the UAE context, suggesting that family-inclusive educational interventions could effectively promote earlier palliative care access in tertiary hospital settings (33, 35).

Pain control emerged as a central component of patient experience (Theme 2). Most participants reported adequate access to analgesia within hospital settings; however, regulatory restrictions and pharmacy access outside hospitals created delays, particularly medications such as tramadol. Similar barriers have been widely reported across the region, including limited opioid availability, regulatory constraints, and fear of addiction (36).

While participants generally expressed satisfaction with symptom relief, concerns regarding opioid use and side effects were evident. Some patients and caregivers reported fears of hallucinations, dizziness, and potential overmedication, highlighting the importance of reassurance and clear communication from healthcare teams. These findings align with regional literature (37) that describes several factors contributing to the lack of access to palliative care services. These include healthcare professionals’ limited knowledge (38), restrictive laws and regulations around opioid access, regulatory barriers (39, 40), and misconceptions or lack of understanding about opioids (41).

Importantly, our findings reinforce that access alone is insufficient, as improved pain control also requires structured education for healthcare providers, patients, families, and policymakers (36). The data also highlight the need for balanced communication strategies that ensure both effective symptom relief and patient safety concerns are addressed.

Cultural expectations and family dynamics significantly shaped decision-making processes (Theme 3). Some participants described family resistance to treatment plans or preferences for seeking care abroad, reflecting tensions between cultural norms and clinical recommendations. These findings are consistent with literature from the Middle East and North Africa (MENA) region highlighting that misconceptions about palliative care, along with sociocultural barriers such as beliefs about death and dying, further complicate the situation (42, 43). For example, the culture in the region has long-standing fatalistic beliefs about death (42, 44), which can reduce public demand for palliative services and sometimes lead to resistance even when they are offered.

Financial considerations were generally mitigated by insurance coverage, which facilitated access to care for most participants. However, a proportion of participants reported uncertainty regarding financial support, suggesting variability in awareness or navigation of the insurance system. These findings are consistent with previous reports indicating that coverage for palliative care varies significantly, and it is challenging to clearly define which services are included as part of a comprehensive palliative care approach (45).

Within the UAE context, where healthcare financing is a mix of public insurance mandates and private coverage systems, variability in reimbursement for palliative care services may influence access and continuity of care. Historically, medical insurance providers have not included coverage for palliative care services in their policies. However, this has changed with the introduction of specific diagnosis-related group (DRG) codes for palliative care, which now covers both inpatient and outpatient consultations (46).

Overall, participants reported positive experiences with palliative care services, particularly in relation to multidisciplinary team involvement, symptom relief, and improvements in quality of life (Theme 4). Some participants described seeking care across multiple international healthcare systems before receiving effective symptom management in the UAE, highlighting the growing capacity of local services to deliver care that is aligned with international standards.

In a few international studies, symptom control (including pain management) and end-of-life care (EoLC) were the most common reasons for referrals to the service, reflecting trends observed by hospital palliative care teams in Europe and Japan (47, 48). However, unlike many international studies, our findings emphasize the importance of local service accessibility and continuity of care within the UAE healthcare system, particularly after fragmented care experiences abroad.

Although spiritual care was not a dominant theme in this study, its importance has been widely documented in palliative care literature as a core component of holistic care (47), suggesting that this area may warrant further exploration in the UAE context.

Participants highlighted key areas for improvement, particularly in communication, staffing, and responsiveness (Theme 5). Language barriers, particularly the limited availability of Arabic-speaking staff or interpreters, were identified as a major challenge affecting patient understanding and satisfaction. This is consistent with evidence showing that language barriers are a key cause of miscommunication between medical providers and patients and negatively affect the quality of healthcare services and patient satisfaction. Many healthcare institutions rely on interpreter services, which can increase both the cost and duration of treatment visits (49).

In addition, participants also identified gaps in staffing, particularly the need for a broader range of palliative care specialists and quicker nurse response times during night shifts. Participants highlighted these issues, suggesting that increased specialization and improved responsiveness could enhance care quality. These findings align with longstanding regional challenges documented in the MENA region. Shamieh and Jazieh (36) noted the shortage of palliative care experts and manpower, including physicians, nurses, medical social workers, and religious advisors, as well as the lack of interdisciplinary teams, as two major barriers to the provision of palliative care. More recently, Nijhawan et al. (5) highlighted that in the UAE there is a shortage of trained palliative care specialists and that providing palliative care, particularly at the end of life, is limited by a lack of suitably trained professionals. Similar to these reported barriers, our participants described insufficient specialist availability and delayed nursing responses, especially during night shifts. However, while prior studies emphasize system-level workforce shortages, our findings provide patient-level evidence of how these gaps directly affect perceived care quality and responsiveness in daily clinical practice.

Improving staffing capacity, increasing specialization in palliative care, and enhancing real-time communication were identified as key priorities. Addressing these gaps may significantly improve patient and caregiver experiences in tertiary oncology settings in the UAE.

Although participants expressed overall satisfaction with palliative care services, their narratives highlight critical areas for improvement, particularly in awareness, communication, cultural responsiveness, and system efficiency. These findings align with international evidence that cancer patients experience multidimensional suffering encompassing physical, psychological, social, and spiritual domains (36).

Collectively, this study underscores that while palliative care services in the UAE are developing and delivering positive outcomes, further efforts are needed to enhance early awareness, strengthen communication strategies, address cultural considerations, and improve workforce capacity. Strengthening these areas will be essential to ensuring equitable, patient-centered palliative care delivery in the UAE and in similar transitional healthcare systems.

## Limitations

The major strength of this study lies in its novelty in exploring both caregivers’ and patients’ insights into palliative care, addressing a significant gap in the literature, which has predominantly focused on healthcare providers. To our knowledge, this is among the first qualitative studies in the UAE to examine the experiences of cancer patients and their caregivers.

The main limitations of this study include the small sample size (N = 10) and its single-center design in a tertiary hospital in the UAE, which may limit generalizability. However, despite these limitations, the findings offer valuable insights that may be transferable to similar multicultural, resource-rich healthcare settings where palliative care services are developing within complex cultural contexts.

Although data saturation was reached after 10 interviews with no new themes emerging, recruitment through the clinical team may have introduced selection bias. Reliance on field notes rather than audio recordings may have reduced data richness, and member checking was not feasible due to patient vulnerability. Nevertheless, methodological rigor and trustworthiness were maintained through consistent data collection procedures, careful thematic analysis, and reflexive engagement with the data.

Despite these constraints, the study provides valuable preliminary insights into cancer patients’ experiences and highlights context-specific palliative care challenges in the UAE. Future multi-center research with larger, more diverse samples and audio-recorded interviews is recommended to strengthen the findings and improve generalizability across the UAE and the broader MENA region.

## Conclusion

This study provides context-specific insights into the role of palliative care in improving quality of life for patients with advanced cancer within the UAE. While participants generally reported positive experiences, particularly regarding multidisciplinary teams and symptom management, important gaps remain in public awareness, provider–patient communication, and timely access to services. Importantly, the findings highlight how palliative care experiences are shaped not only by healthcare delivery structures but also by the sociocultural and regulatory context of the UAE. Improving palliative care in the UAE requires more than service expansion; it calls for culturally sensitive communication, enhanced public and professional education, and policy approaches tailored to the local context. Strengthening integration within oncology services and aligning care with sociocultural realities are essential to achieving equitable, patient-centered outcomes. Evaluating long-term outcomes of palliative care interventions in the UAE is essential to assess their impact on quality of life and symptom management. Policymakers and researchers should focus on integrating palliative care into national frameworks while addressing cultural, financial, and logistical barriers, including insurance constraints and workforce shortages.

## Data Availability

The original contributions presented in the study are included in the article. Further inquiries can be directed to the corresponding author.
